# Julie Denekamp April 10th 1943–June 6th 2001

**DOI:** 10.1054/bjoc.2001.2063

**Published:** 2001-09

**Authors:** Fiona Stewart

**Affiliations:** Experimental Therapy (H6), The Netherland Cancer Institute, Plesmanlaan 121, Amsterdam, CX, 1066, The Netherlands


British Journal of Cancer (2001) 8 5(7), 937–938
© 2001 Cancer Research Campaign
doi: 10.1054/ bjoc.2001.2063, available online at http://www.bjcancer.com

Obituary

### Julie Denekamp
### April 10th 1943–June 6th 2001

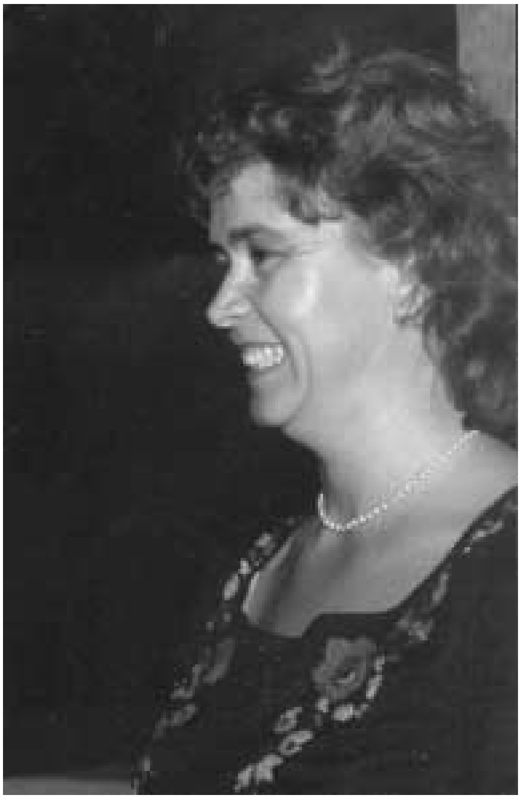

On 6th June 2001, Julie Denekamp died, aged 58, after a 2 year struggle with inflammatory breast cancer. After her diagnosis, Julie and her husband Bo Littbrand remained at their home in Sweden, where Julie continued to supervise and direct her Translational Research Group at Umea University. She was still correcting manuscripts for her students (with that famous red pen) until one week before her death! Julie and Bo also tried to spend as much time as possible at their second home in Cornwall, England. They were en route to Cornwall, visiting Julie's mother in Cwmbran, Wales, when Julie died after having a happy and peaceful few days with her family. Unfortunately, she never saw her first grandchild, Cèire Julianne, who was born a few days later to her daughter Liz and Stephen Fowley.

Julie was truly one of the driving forces in the radiobiology community. She made a big impression on all who met her, with her spirited and enthusiastic approach to both her professional and personal life. One of Julie's hallmarks was her love of a good debate. Since Julie was blessed with an exceptionally sharp mind and superb didactic skills, this meant that she had often won the debate before the rest of us had sorted out what the main issues were! Participating in scientific discussions with Julie was an exhilarating and educational experience and several generations of radiobiologists, both older and younger than she, have benefited enormously from her insight, knowledge and encouragement.

Another hallmark was her courage and integrity. Julie would fight tirelessly to search out a scientific truth or to defend a moral principle or person in whom she believed. She was always committed to the truth, no matter if this went against accepted dogma. This sometimes made her own life difficult and led to conflicts with the establishment but she never took the easy way out.

Julie graduated with a first class honours degree in zoology and botany from London University in 1964. She was then appointed by Jack Fowler to work as his research assistant at the MRC Postgraduate School, Hammersmith Hospital, London. In addition to the work Julie did with Jack, on fractionation effects of radiation in normal tissues and tumours, Julie began her own research on tumour cell kinetics. This resulted in her PhD thesis in 1968. After completing her thesis, Julie went with her first husband, Stan Field, and baby daughter Caroline to Stanford, for a 1 year post-doctoral period. Shortly after returning to London, their second child, Elizabeth, was born. In 1971 Julie moved with Jack Fowler, Adrian Begg, Peter Sheldon and Susan Harris from Hammersmith to the Gray Laboratory, where Jack was appointed as Director.

By the time I became Julie's research assistant, in the summer of 1973, she already had a reputation as an excellent experimental scientist and her lecturing and debating skills were legendary. Julie could initially seem somewhat intimidating, with her direct and sometimes confrontational approach to scientific discussions. How many of us have experienced that quizzical raised left eyebrow which seemed to say 'are you absolutely sure about that'? However, it soon became apparent that Julie took great delight in discussing and analysing scientific results and that she was only interested in looking critically at the data, not the experimenter or discussant. Julie was, in fact, an inspirational teacher and mentor. She had an enviable ability to assess and assimilate data and to present them in a clear and logical fashion. She also managed to instil enthusiasm and excitement into her team and was always loyal and supportive. Cancer research was more than a job to Julie; it was a passion.

Julie worked at the Gray Lab for almost 25 years. In 1977 she was made Head of the Radiobiology Applied to Therapy Section, and in 1988 she was appointed as the Institute Director, succeeding Jack Fowler. During this period she made many seminal contributions in the field of oncology and in 1980 she was awarded the prestigious DSc degree. Some of her most significant contributions include her work on time factors and compensatory proliferation after fractionated radiotherapy, modifiers of the radiation response to exploit differences between tumours and normal tissues, e.g. hyperfractionation, radiosensitisers, radioprotectors and hyperthermia, and her pioneering work on targeting the tumour vasculature as a novel form of cancer treatment. It was Julie who showed that the proliferation rate of endothelial cells in tumours was about 100 times that in normal tissues. From the moment she had observed this difference, in the early 1980s, Julie realised that this represented an opportunity for specific tumour targeting and she redirected a large part of her research programme to this end. Over the last few years, vascular targeting has become widely recognised as one of the most promising new approaches for cancer therapy. Other examples where Julie was instrumental in initiating new clinical protocols based on results from the laboratory (true 'translational research') are the accelerated, hyperfractionated, radiotherapy schedule CHART and, more recently, the ARCON trials, which combine accelerated radiotherapy to overcome tumour proliferation, with carbogen and nicotinamide to overcome tumour hypoxia.

In addition to her scientific achievements at the Gray Lab, Julie was responsible for initiating numerous building, refurbishing and upgrading projects. These included conversion of the derelict Art Nouveau hospital chapel into the present-day, stylish Fowler Scott Library. Since the funds available for this conversion were limited, Julie motivated the entire lab to 'volunteer' to do much of the refurbishing themselves. It was typical of Julie that she imbued her own, personal style on such a project and motivated an entire team to willingly give their time to achieve the goal. I'm sure that this will strike a chord with the many visiting workers who passed through the Gray Lab while Julie was there. They will all have experienced her infectious enthusiasm and generosity, both in scientific exchanges and on a personal level.

By the end of the 1980s Julie had a new love in her life, Bo Littbrand, who was then the Dean and Head of Radiotherapy at Umea University in Sweden. Julie and Bo were married in June 1989 and many of her friends and colleagues were privileged to share their happiness on that beautiful summer's day. For 5 years after their marriage. Julie continued as Director of the Gray Lab, but increasing struggles with CRC Head Office over funding, organisational structure and future directions took their toll. In 1994 Julie left the Gray Lab and moved to Sweden so that she and Bo could spend more time together. No doubt Julie also thought she would be free of administrative hassle and fights over resources!

In 1995 Julie was appointed as Professor of Radiobiology in the Oncology Department at Umea University. Together with Bo, she built up a new Translational Research Group, with a mixture of clinicians, physicists and biologists. This period of Julie's life was also very productive, despite ill health and recurring conflicts with established groups within the university over space and resource allocations. During her time in Umea, Julie and her group focused on analyses of clinical data, on long-term morbidity after radiotherapy for breast cancer, proliferation patterns in colorectal cancer, and on modelling the impact of tumour hypoxia on treatment. Julie was particularly excited about the possibility of exploiting chronic tumour hypoxia for radiosensitisation, if these metabolically starved cells become repair incompetent. This is yet another example of how Julie was always prepared to challenge established dogma and look at experimental data in a new light.

In addition to her research activities in Umea, Julie was very active in organising teaching courses, workshops and conferences. In all of these activities Julie's personal style and flair was apparent. Not only were the courses of a high scientific and educational value, they also introduced the participants to local Swedish culture, cuisine and nature. Julie made sure that the intensive scientific programme was balanced by an excellent social programme with picnics at local beauty spots. She and Bo would also entertain course participants and lecturers at their own home in Innertavle.

These are some of the reasons that Julie has had such a big impact on the lives of all who knew her and worked with her. Julie will be missed for her warmth, humour and lively friendship, as much as for her very considerable scientific contributions to the field. Julie was special, and she will continue to be remembered with affection and respect for many years to come.

*Dr Fiona Stewart*
*Experimental Therapy (H6)*
*The Netherland Cancer Institute*
*Plesmanlaan 121*
*1066 CX Amsterdam*
*The Netherlands*

 © 2001 Cancer Research Campaign

